# Perceived stress and family adaptability in head and neck cancer patients receiving radiotherapy: the chain-mediated effect of social support and family resilience

**DOI:** 10.3389/fpsyt.2024.1488196

**Published:** 2025-01-17

**Authors:** Xiaoru Li, Yu Zhu, Hongwei Wan

**Affiliations:** ^1^ Department of Nursing, Shanghai Proton and Heavy Ion Center, Fudan University Cancer Hospital, Shanghai, China; ^2^ Shanghai Key Laboratory of Radiation Oncology, Shanghai Proton Heavy Ion Hospital, Shanghai, China; ^3^ Shanghai Engineering Research Center of Proton and Heavy Ion Radiation Therapy, Shanghai Proton Heavy Ion Hospital, Shanghai, China

**Keywords:** head and neck cancer, perceived stress, social support, family resilience, family adaptability, chain mediating effect

## Abstract

**Objective:**

Patients with head and neck tumors undergoing radiotherapy are burdened with a variety of disease-related stressors that may affect their family adaptability. The aim of the present study was to investigate the relationship between perceived stress and family adaptability in patients with head and neck tumors and to analyze whether social support and family resilience play a mediating role in this relationship.

**Methods:**

The convenience sample approach was utilized to recruit 316 patients with head and neck tumors who received radiation. Self-developed general information questionnaires, the Chinese Perceived Stress Scales, Social Support Rating Scale, the Shortened Chinese Version of the Family Resilience Assessment Scale, and Family Adaptability Scale were used to collect data. Bootstrap methods to analyze independent and chained mediation effects between variables.

**Results:**

The research participants had a mean age of 43.63 ± 12.78 years, were mostly male (61.7%), married (85.8%), had a university education (51.6%), were uninsured (50.9%), had ear, nose, and throat tumors (56.3%), and had an illness duration of 1-6 months (43.4%). The findings of the chain mediation effect research indicate that the direct negative effect of perceived stress on family adaptability (-0.163) accounted for 45.63% of the overall effect (-0.355), while the indirect effect (-0.194) accounted for 54.37%. Perceived stress independently mediated family adaptability through social support (effect: -0.062) and family resilience (effect: -0.080), with the independent mediator effect accounting for 32.12% and 41.45% of the indirect effect, respectively, and chain-mediated mediation of social support and family resilience, with the chain effect (-0.051) accounting for 31.30%.

**Conclusion:**

Perceived stress in patients with head and neck cancer receiving radiotherapy directly or indirectly negatively affects family adaptability. Clinical staff should meet the patient’s health care service needs while also utilizing the family’s internal and external resources to reduce disease-related stress and improve family adaptability.

## 1 Introduction

Head and neck tumors with predominantly squamous epithelial malignant lesions are radio-sensitive, so radiotherapy is the main treatment, and about 75% of patients receive radiotherapy (1). Head and neck tumors are in close proximity to salivary glands, the larynx, oral and pharyngeal mucosa, cranial nerves, and other important tissues and organs, and radiotherapy inevitably leads to toxic side effects such as oral mucositis, dysphagia, hoarseness, clenching of the teeth, hearing loss, etc., which lead to physical dysfunction of the patients in terms of vision, hearing, communication, and eating (1, 2). Head and neck tumors are considered the most emotionally traumatic. Treatment leads to facial disfigurement and functional impairment, as well as social difficulties due to the long-term course of the disease, with a cascade of effects on the patient’s self-image, relationships with partners, and social and sexual functioning, suffering from cancer-related shame, psychological distress, and disturbed body image (1, 2). Also, patients experience family tensions and are vulnerable to psychiatric comorbidities (especially anxiety and depression) due to concerns about health, work, and finances (3).

In addition, head and neck tumors directly affect the physical and mental health and functioning of family members (4). Family members reported physical and psychological discomforts, such as anxiety and depression, fatigue, sleep disorders, weight loss, loss of appetite, and headaches, as well as a decline in social functioning and quality of life, which limits the use of family resources and severely hampers the family’s healthy development (5–7). Some families overcome disease stress and adapt to life changes by continuously adjusting their mode of operation during the process of tumor diagnosis and radiotherapy (8), possibly because good family adaptability strengthens family cohesion, provides access to more support for cancer patients (9), and improves patients’ ability to cope with their illnesses. Therefore, it is critical to identify factors of family adaptability and discover the potential mechanisms by which these factors affect family adaptability.

Patterson’s Family Adjustment and Adaptation Response (FAAR) model emphasizes family members’ adaptation by balancing family demands with family capabilities and interacting with family meanings (10). Family demands are the various long-term, short-term, anticipated, or unanticipated stressful events that families face; family capacity is the psychosocial resources that families possess and the coping behaviors they adopt; family meaning refers to the family’s assessment of family needs and family capacity, as well as to the worldview of family members and their identification with their family identity (10). Head and neck tumors provide family members with long-term exposure to stressful events, which can easily lead to negative perceptions and evaluations of the disease and affect the patient’s ability to adapt to stress adjustments (7, 11). Cohen proposed that perceived stress refers to the degree to which an individual assesses a stimulus event as stressful, implying that the stressful impact of an objective stressful event on an individual is determined by the event’s subjective interpretation and perception (12). Previous research has found that perceived stress negatively impacts family adaptability among family caregivers of young and middle-aged Chinese cancer patients (13).

Psychosocial resources play an important role in maintaining family capacity (10). Social support is what individuals receive in their social networks, including emotional support, information support, and material support (14). On the one hand, social support alleviates perceived disease stress in cancer patients and has a positive effect on improving quality of life (i.e., social activities, physical functioning, and mood) (15). According to the stress buffer model, during periods of acute stress, social support changes the individual’s assessment of stressful events and acts as a protective buffer against negative effects (16). Previous studies have found that emotional support from family members, informational support from friends (17), and professional support from healthcare professionals (15) reduce perceived stress in cancer patients. On the other hand, social support plays an important role in improving family adjustment in oncology patients (18), probably because it helps to enhance emotional ties among family members, reduce the burden of family caregiving (19), and improve family functioning (20). Empirical studies have shown that internal and external social support within the family positively predicts family adaptability in Chinese patients with primary liver carcinoma (21). This study postulates that social support may play a mediating role between perceived stress and family adaptability.

Coping is considered part of the family’s capacity (10). It is necessary for families in distress to develop resilience beforehand in order to maintain a normal life trajectory (22). Family resilience refers to the ability of a family to effectively cope with and adapt to a new mode of functioning after an adverse event (22), and it is a potentially positive strength of the family that facilitates the development of positive psychological qualities among family members to cope with stressful events (23). It was found that perceived stress was negatively correlated with family resilience in teenagers with a cancer-suffering parent, especially when the parent’s condition deteriorated or they underwent radiation therapy, and the adolescents showed a stronger stress response, suggesting that patients with a higher perception of stress had correspondingly lower levels of family resilience (24). Family resilience plays an important role in achieving family adaptation, possibly through mechanisms that activate protective resources at the individual (education of family members, income), family (communication, cohesion), and social (health care and educational services) levels to promote family resilience, cope with and buffer against stressful events, and contribute to the family’s survival and emergence during major crises (10, 25). Looking at families of cancer patients (18), dementia patients (26), and sick children (27, 28) showed that family resilience independently and positively predicted family adaptability. Previous research has demonstrated that family resilience in cancer patients mediates the relationship between perceived stress and family adaptability (13). Therefore, it is hypothesized that family resilience may play an independent mediating role in perceived stress and family adaptability.

The FAAR model emphasizes the interaction of family needs and family capabilities to maintain family equilibrium. When a family crisis exceeds the family’s capacity and resources and the imbalance persists, it can lead to significant changes in family structure, interaction patterns, etc., affecting family adaptability and even leading to a greater crisis (10). However, not all cancer patients report inadequate adaptive capacity (20, 26). Family resilience and social support are potential internal and external resources for families that contribute to the level of family resilience (18, 21, 28). Family coping strategies, such as giving positive meaning to risk events, adopting positive coping strategies, maintaining clear family boundaries, improving communication skills of family members, fostering family flexibility and cohesion, and actively integrating into the community and seeking professional support, are conducive to enhancing family resilience and playing a protective role for the family (10, 22). Studies have shown that social support received by families of lung cancer patients significantly and positively affects family resilience (19), and this result is consistent with Walsh’s (22) family resilience framework, which proposes that social resources are an important protective factor for family resilience. Furthermore, perceived stress is negatively correlated with social support (17), and family resilience positively predicts family adaptability (18). We propose the hypothesis that social support and family resilience may serve as chain mediators between perceived stress and family adaptability.

Although the relationship between perceived stress, social support, family resilience, and family adaptation has been examined separately, no relevant research has been found on the chain-mediated effects of social support and family resilience in perceived stress and family adaptation. With the FAAR model, stress buffer model, and family resilience framework, the current study sought to determine whether social support and family resilience mediate the relationship between perceived stress and family adaptability in head and neck cancer patients receiving radiotherapy. To inform the development of family-oriented interventions that are consistent with Chinese culture.

## 2 Materials and methods

### 2.1 Participants

This was a cross-sectional study conducted from December 2021 to December 2022, using convenience sampling to recruit patients with head and neck tumors undergoing radiotherapy at Shanghai Proton Heavy Ion Hospital, China. Inclusion criteria: (1) age ≥18 years; (2) diagnosed with head and neck tumors; (3) no communication difficulties, able to understand and answer questions, and complete the questionnaire independently or with the assistance of the investigator; (4) receiving radiotherapy only; (5) informed consent and voluntary participation in this study. Exclusion criteria: (1) history of mental illness; (2) comorbidity with other vital organ diseases; (3) participation in other similar studies during the same period. The dependent variable was family adaptability; the sample size was calculated using relevant literature (18) and the sample size estimation formula for cross-sectional studies: n=(t_α/2_S/δ)^2^ (29), with a standard error of S=8.61, a test level α=0.05, and a tolerance error δ=1, resulting in n=285. Considering a 10% loss due to follow-up and sampling error, the final sample size was determined to be at least 314 cases.

### 2.2 Measures

#### 2.2.1 Demographic and disease-related variables

The questionnaire created by the research team based on the literature review was used to collect patient demographic information such as age, gender, marital status, educational level, annual household income, commercial insurance, tumor site, tumor stage, disease duration, and radiation therapy regimen. Radiotherapy costs at Shanghai Proton Heavy Ion Hospital are entirely self-funded or reimbursed by commercial insurance, so the investigation into medical cost reimbursement was conducted using commercial insurance.

#### 2.2.2 Perceived stress

The Chinese Perceived Stress Scales (CPSS) were used to measure perceived stress. The scale was developed by Cohen (12), and the Chinese version was revised by Yang et al. (30). The scale has 14 items and two dimensions: a sense of tension and a sense of loss of control. The Likert 5-point scale was used, with scores from 0 to 4 indicating “never, occasionally, sometimes, often, always.”. Items 4-7, 9-10, and 13 are reverse scored, while items 1-3, 8, 11-12 are forward scored, for a total score of 0-56. The higher the score, the higher the stress level. The Cronbach’s alpha coefficient for the total scale was 0.78 (30). The Cronbach’s alpha coefficient in this study was 0.856.

#### 2.2.3 Social support

Xiao (31) developed the Social Support Rating Scale (SSRS), which consists of 10 items, to measure social support. The scale includes three dimensions: objective support, subjective support, and support utilization. The scoring method is as follows: For the 1st to 4th and 8th to 10th entries, each entry has 4 options, and only one of them can be chosen, and which option is chosen counts for how many scores; the 5th entry consists of five questions, each of which adopts the scoring method of 1-4 (which means “none, very few, general, and full support, respectively); the 6th to 7th entries: Choosing “no source” scores 0 points, and if “the following sources” is chosen, how many scores will be given to how many options are chosen? The total score was 12-66, with higher scores indicating higher levels of social support. The total entry score for Cronbach’s alpha coefficient was 0.825-0.896 (31). The Cronbach’s alpha coefficient in this study was 0.836.

#### 2.2.4 Family resilience

The level of family resilience was assessed using a shortened Chinese version of the family resilience assessment scale (FRAS-C) (32). Sixbety (33) compiled the source scale, which was later revised in Chinese by Chinese scholar Li (32). FRAS-C consists of 32 items with 3 dimensions: family communication and problem solving; utilizing social resources; and making a positive outlook. The scale is rated on a 4-point Likert scale, with scores ranging from 1 to 4 indicating “strongly disagree” to “strongly agree” and a total score ranging from 32 to 128, with higher scores indicating greater family resilience. The overall scale Cronbach’s alpha coefficient was 0.95 (32). Cronbach’s alpha coefficient was discovered to be 0.96, with subscale Cronbach’s alpha values ranging from 0.69 to 0.94 in Chinese breast cancer patients (23). In this investigation, Cronbach’s alpha was 0.958.

#### 2.2.5 Family adaptability

Family adaptability was measured using the Family Adaptability Scale (FAS), a subscale of the Family Adaptability and Cohesion Scale Olson et al. (34) designed the source scale, while Fei et al. (35) amended the Chinese version, which has 14 items. A Likert 5-point scale was used, with 1-5 indicating “not always” and a total score of 14-70. Higher scores indicate stronger family adaptation, with a Cronbach’s alpha coefficient of 0.73 and retest reliability of 0.91 (35). The Cronbach’s alpha coefficient for this study was 0.814.

### 2.3 Procedures

Following the GCP principles and the Declaration of Helsinki, Shanghai Proton Heavy Ion Hospital’s Ethics Committee approved this study (Ethics No. 2202-53-03). First, communicating with the Director of Nursing at the hospital to explain the purpose of this survey. Connections were made to obtain approval from the head nurse of the head and neck oncology unit. Second, two staff members were recruited and provided with data collection training to ensure their adequate expertise. Lastly, the staff used a uniform language to introduce the purpose, content, and significance of the study to the subjects and began distributing the questionnaires after obtaining informed consent from the subjects, which were completed by the patients themselves. To ensure patient privacy, the questionnaires were completed in the departmental conversation room. In principle, the patient fills out the scale independently; however, if the patient suffers vision loss due to illness, the researcher may read the inputs aloud verbatim to aid with completion. It took 15-20 minutes to do all of the surveys. The surveys were completed on the spot, and any omitted or inaccurate information was promptly reviewed and added to the patients’ records. A total of 345 questionnaires were delivered, and after screening and removing 29 invalid questions that were filled out incorrectly, 316 valid questionnaires were retrieved, yielding an effective recovery rate of 89.3%.

### 2.3 Data analysis

All the data analysis and processing were completed using IBM SPSS 24.0 software. Harman’s one-way test method of unrotated principal component factor analysis of all scale measurement entries showed that the first common factor explained <40% of the total variance in variance, indicating that there was no significant methodological bias.

Socio-demographic and disease-related characteristics, as well as variables of interest, were described using descriptive statistics. Categorical variables were described using frequencies and percentages. Measures of interest were analyzed for normality using the Kolmogorov-Smirnov test, with conformity to normal distribution reported as mean (*M*) and standard deviation (*SD*), and non-conformity expressed as median and quartile. Independent sample t-tests and one-way ANOVA were used to test for differences in family adaptability between socio-demographic factors and disease characteristics, and the least significant difference performed *post-hoc* tests for groups where differences existed. Pearson correlation analyses were used to see if there was any correlation between perceived stress, social support, family resilience, and adaptability. In multiple linear regression analyses, independent factors included socio-demographic and illness characteristics, perceived stress, social support, and family resilience, with family adaptability serving as the dependent variable.

The direct and indirect effects of perceived stress on family adaptability were examined using bootstrap analyses with 5,000 bootstrap samples. Using Process Model 6, developed by Hayes (36), examine the chain mediation model and determine whether the indirect effects of each mediator are independent. The mediating effect was significant if the 95% bias-corrected confidence interval did not include zero. A value of *P* < 0.05 (two-tailed) was considered statistically significant.

## 3 Results

### 3.1 Common method biases tests

There were a total of 70 entries for all measurement scales. The results of Harman’s one-way test showed that there were 16 common factors with eigenvalues greater than 1. The first common factor had an eigenvalue of 18.33, and the total variance of the explained variance was 26.19 percent, which was less than the critical value of 40 percent. Therefore, there is no significant common method bias in this study.

### 3.2 Descriptive statistics

#### 3.2.1 Descriptive statistics and variation analysis

**Table 1** presents the socio-demographic and disease-related characteristics of the participants. The mean age of the 316 patients with head and neck tumors who underwent radiotherapy was 43.63 ± 12.78 years (age range 18-77 years). The findings of the independent sample t-test and one-way ANOVA revealed that education level, annual household income, and disease duration were significantly associated with family adaptability. Other characteristics of the participants are detailed in **Table 1**.

**Table 1:** Socio-demographic and disease characteristics of patients with head and neck tumors receiving radiotherapy (*n* = 316).

Variable	*n* (*%*)	Family Adaptability (*M* ± *SD*)	*t*/*F*	*P*
Age (year)			0.334	0.716
18~40	144 (45.6)	50.20 ± 7.13		
41~65	155 (49.0)	50.88 ± 7.52		
≥66	17 (5.4)	50.18 ± 8.16		
Gender			-0.811	0.418
Male	195 (61.7)	50.27 ± 7.50		
Female	121 (38.3)	50.96 ± 7.15		
Marital status			1.443	0.238
Married	271 (85.8)	50.25 ± 7.51		
Unmarried	39 (12.3)	52.08 ± 5.64		
Widowhood	6 (1.9)	53.17 ± 9.62		
Educational level			9.859	<0.001
Primary school or lower	25 (7.9)	44.04 ± 8.06		
Middle school	101 (32.0)	49.64 ± 7.81		
University	163 (51.6)	51.96 ± 6.68		
Master or higher	27 (8.5)	51.22 ± 5.16		
Annual household income (yuan)			5.326	0.001
<15 thousand	90 (28.5)	48.02 ± 8.09		
15~30 thousand	119 (37.7)	51.15 ± 6.37		
30~50 thousand	75 (23.7)	52.08 ± 7.44		
>50 thousand	32 (10.1)	51.66 ± 7.03		
Commercial insurance			1.106	0.269
Yes	161 (50.9)	50.98 ± 7.50		
No	155 (49.1)	50.06 ± 7.22		
Tumor site			0.548	0.650
Base of the skull	72 (22.8)	49.90 ± 7.69		
Ear nose and throat	178 (56.3)	50.53 ± 7.82		
Oral and maxillofacial cavity	56 (17.7)	50.95 ± 5.34		
Neck	10 (3.2)	52.80 ± 6.61		
Tumor stage			0.833	0.476
I	47 (14.9)	49.64 ± 7.33		
II	72 (22.8)	50.82 ± 6.31		
III	93 (29.4)	51.35 ± 7.56		
IV	104 (32.9)	50.00 ± 7.87		
Disease duration (months)			7.315	0.001
<6	137 (43.4)	52.28 ± 7.16		
6~12	86 (27.2)	49.59 ± 7.19		
>12	93 (29.4)	48.83 ± 7.33		
Radiation therapy regimen			1.669	0.174
Proton	64 (20.3)	51.44 ± 7.29		
Heavy ion	92 (29.1)	49.12 ± 8.01		
Proton + Heavy Ion	56 (17.7)	50.84 ± 7.06		
Heavy Ion + Photon	104 (32.9)	51.06 ± 6.87		

*M*, mean; *SD*, standard deviation. *t*, t-test; *F*, One-way ANOVA.

*P* < 0.05 was considered statistical significance.

#### 3.2.2 *Post hoc* tests to analyze differences

**Table 2** shows *post-hoc* tests comparing the levels of family adaptation of head and neck cancer patients in different groups based on education, annual household income, and disease duration, which revealed that all three variables were significantly associated with family adaptation and thus used as covariates in the chained mediation model.

**Table 2:** *Post hoc* tests analyzing differences in family adaptation in terms of education level, annual household income, and disease duration.

Variables	Variables(I)	Variables(J)	Mean difference(I-J)	*P*
Educational level	Primary school or lower	Middle school	-5.604	<0.001
		University	-7.923	<0.001
		Master or higher	-7.182	<0.001
	Middle school	University	-2.320	0.010
		Master or higher	-1.579	0.304
	University	Master or higher	0.741	0.615
Annual household income (yuan)	<15 thousand	15~30 thousand	-3.129	0.017
		30~50 thousand	-4.058	0.006
		>50 thousand	-3.634	0.108
	15~30 thousand	30~50 thousand	-0.929	0.939
		>50 thousand	-0.505	0.999
	30~50 thousand	>50 thousand	0.424	1.000
Disease duration (months)	1~6	6~12	2.684	0.007
		>12	3.449	<0.001
	6~12	>12	0.765	0.479

*P* < 0.05 was considered statistical significance.

### 3.3 Correlations among main variables

Pearson correlation was used to analyze the correlation between perceived stress, social support, family resilience, and family adaptability, and the results are shown in **Table 3**. There was a significant negative correlation between perceived stress and social support, family resilience, and family adaptability, while there was a significant positive correlation between social support and family resilience, social support and family adaptability, and family resilience and family adaptability.

**Table 3:** Correlation analysis between key variables (*n* = 316).

	Variable	*M*	*SD*	1	2	3	4
1	perceived stress	20.48	7.99	1			
2	social support	42.54	7.41	-0.408^**^	1		
3	family resilience	96.93	12.37	-0.445^**^	0.582^**^	1	
4	family adaptability	50.53	7.36	-0.444^**^	0.511^**^	0.616^**^	1

*M*, mean; *SD*, standard deviation.

^**^ *P* < 0.01.

### 3.4 Multiple linear regression analysis

The covariate covariance diagnostic revealed that independent variables had a tolerance >0.1 and VIF <10.0, indicating no multicollinearity. Multiple regression analysis revealed that, after controlling for covariates (educational level, annual household income, and disease duration), perceived stress, social support, and family resilience all had a significant influence on family adaptation in patients with head and neck tumors (**Table 4**).

**Table 4:** Multiple Linear regression of factors associated with family adaptation.

Factors	Unnormalized coefficients		Standardized Coefficients	*t*	*P*
	*B*	*SEs*	*Beta*		
(Constant)	22.655	3.599		6.294	<0.001
Educational level	0.403	0.446	0.041	0.904	0.367
Annual household income	-.0148	0.347	-0.019	-0.428	0.669
Disease duration	-0.500	0.383	-0.057	-1.304	0.193
Perceived stress	-0.162	0.045	-0.176	-3.624	<0.001
Social support	0.191	0.053	0.192	3.585	<0.001
Family resilience	0.240	0.035	0.403	6.860	<0.001

*R* ^2^ = 0.443, Δ*R* ^2^ = 0.432, *F*=40.940, *P*<0.001.

### 3.5 Chain mediation model

**Figure 1:**
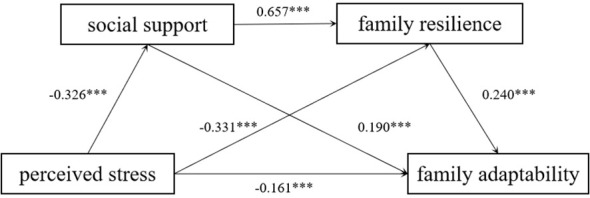
The chain mediating effect of social support and family resilience. ^***^ *P* < 0.001.

Chain mediation effects were tested using PROCESS 4.0 Model 6. **Table 5** shows the regression analysis results. In Model 1, perceived stress significantly negatively predicted social support (*b* = -0.326, *P*<0.001); in Model 2, perceived stress significantly negatively predicted family resilience (*b* = -0.331, *P*<0.001), and social support significantly positively predicted family resilience (*b* = 0.657, *P*<0.001); in Model 3, perceived stress significantly negatively predicted family adaptability (*b* = -0.161, *P*<0.001), and both social support (*b* = 0.190, P<0.001) and family resilience (*b* = 0.240, *P*<0.001) significantly positively predicted family adaptability. In summary, social support and family resilience produce chain-mediated effects between perceived stress and family adaptation (**Figure 1**).

**Table 5:** Regression results of the chain mediating effects model (*n* = 316).

Outcome variable	Predictive variable	*R^2^*	*F*	*b*	*SEs*	*t*	*LLCI*	*ULCI*
Model 1								
social support	perceived stress	0.229	23.157^***^	-0.326^***^	0.047	-6.899	-0.420	-0.233
Model 2								
family resilience	perceived stress	0.478	56.919^***^	-0.331^***^	0.070	-4.742	-0.469	-0.194
	social support			0.657^***^	0.078	8.427	0.503	0.810
Model 3								
family adaptability	perceived stress	0.442	40.940^***^	-0.161^***^	0.044	-3.623	-0.249	-0.073
	social support			0.190^***^	0.053	3.585	0.086	0.295
	family resilience		0.240^***^	0.035	6.859	0.171	0.308

*SEs*, standard error; *LLCI*, Lower limit of the 95% CI; *ULCI*, Upper limit of the 95% CI.

^***^ *P* < 0.001.

**Table 6** presents the results of the chained mediation analysis. The 95% confidence intervals for the total, direct, and indirect effects were found to be non-zero, indicating that perceived stress has a significant impact on family adaptability, directly or indirectly. The standardized direct effect (-0.162) accounted for 45.63% of the total effect (-0.355), while the standardized indirect effect (-0.194) accounted for 54.37%, demonstrating that perceived stress indirectly affects family adaptability in a dominant role. The mediating effect consisted of three pathways, respectively: (1) the effect of perceived stress affecting family adaptability through social support (-0.062) accounted for 32.12% of the standardized indirect effect; (2) the effect of perceived stress affecting family adaptability through family resilience (-0.079) accounted for 41.45% of total indirect effects; and (3) the chain effect of perceived stress affecting family resilience sequentially through social support and family resilience (-0.052) accounted for 26.43% of the indirect effect. In conclusion, the independent mediating effects of perceived stress through social support and family resilience, respectively, and the chain mediating effects they form significantly affect family adaptation, consistent with the hypotheses of this study.

**Table 6:** Total, direct, and indirect effect of perceived stress on family adaptability though social support and family resilience.

Path	Effect	Boot *SE*	Boot LLCI	Boot ULCI	Effect ratio
Total effect	-0.355	0.046	-0.445	-0.264	
Direct effect	-0.162	0.044	-0.249	-0.073	45.63%
Total indirect effect	-0.193	0.032	-0.258	-0.132	54.37%
X→M1→Y	-0.062	0.020	-0.106	-0.025	32.12%
X→M2→Y	-0.080	0.023	-0.130	-0.038	41.45%
X→M1→M2→Y	-0.051	0.013	-0.080	-0.028	26.43%

X = perceived stress; Y = family adaptability; M1 = social support; and M2 = family resilience.

## 3 Discussion

This study analyzes the relationship between perceived stress and family adaptability in patients with head and neck tumors undergoing radiotherapy and also explores whether social support and family resilience have a chain-mediated role. The findings were consistent with the hypotheses that perceived stress directly and negatively affects family adaptability; social support and family resilience partially mediate the relationship between perceived stress and family adaptability, respectively; and social support and family resilience have a chain mediating effect.

The study’s findings revealed that perceived stress has a direct and negative impact on family adaptability in head and neck cancer patients receiving radiotherapy, which is consistent with previous findings (13) that lower levels of perceived stress in caregivers of young and middle-aged cancer patients are associated with better family adaptability. Prolonged and severe stressful events increase family susceptibility, decrease family cohesiveness, and may even lead to family breakup (37), whereas families with high adaptation capacity successfully cope with stress and preserve stable family growth (8). This may be because high levels of family resilience are associated with positive psychological states and coping behaviors in individuals (38) and because family members are close (9) and supportive of each other, which improves their ability to resist stress. Families are responsible for monitoring the patient’s illness, scheduling outpatient follow-up appointments, and giving financial and emotional support to the patient for a long period of time, resulting in significant levels of strain and stress (39, 40). The study’s findings revealed that the direct effect value of perceived stress on family adaptability was -0.162, which has low explanatory power, most likely because family members were not included as respondents in the pair survey. Clinical staff are encouraged to use family-centered treatments to increase the family’s ability to resist and adapt to stress, as well as to assist patients and family members in gaining confidence in dealing with stress and successfully overcoming tough situations (7).

The current study revealed that social support partially mediates the relationship between perceived stress and family adaptability, i.e., lower levels of perceived stress may lead to higher levels of social support, which may have a positive effect on increasing family adaptability. Cancer patients with a high level of social support are more likely to receive guidance and assistance from family members or friends, effectively alleviating the perceived stress of the disease, including disease-induced emotional trauma [e.g., psychological distress (41), anxiety, and depression (42)], physical dysfunction, and limitations in social activities (15). This study found that stress perception explained 22.9% of the variance in social support, suggesting that only a partial influence of social support is captured, but suggesting that patients with head and neck tumors may seek social support to alleviate the stress of their illness. Furthermore, social support is linked to active psychological states [e.g., post-traumatic growth (43), psychological resilience (44)], and coping behaviors (42), in which family members confide in one another’s inner thoughts and develop and implement effective coping strategies to improve family adaptability (20, 22). In the current investigation, patients with head and neck tumors were mostly treated using proton and/or heavy-ion radiography. The high expense of therapy and the unclear prognosis of the condition place a financial and emotional strain on family members, resulting in a persistently stressed atmosphere and weakening family function. Healthcare professionals are an important source of social support for cancer patients. Healthcare professionals and families are important sources of social support for tumor patients. Healthcare personnel should meet the patient’s needs for professional knowledge related to disease treatment and rehabilitation. Family members should seek support from social organizations as much as possible to obtain additional financial and material assistance and, at the same time, spend more time with and care for the patient to create a warm family atmosphere and assist the patient in adapting to the new environment and new role.

Consistent with previous research (13), the results of this study demonstrate that perceived stress can modulate the level of family adaptability through family resilience. On the one hand, family resilience is adversely associated with perceived stress. The higher the perceived stress level, the higher the negative emotions of family members, which can easily lead to a decrease in the frequency of family communications, provoke family disputes, and so limit the realization of the family’s potential strengths (24, 25). In traditional Chinese culture, which highlights the family as a whole and concentrates on family harmony and mutual care, the family’s beneficial response to stressful circumstances promotes family members’ sense of efficacy. Also, when a family member is seen to be protected due to conditions, the family will unconditionally devote time and energy to assisting the patient in addressing the crisis. On the other hand, family resilience predicts family adaptability. Families with high resilience have more available resources and advantageous strengths, which can promote the development of positive psychological qualities in patients (23), allowing them to successfully cope with the stressful stress of the condition and adapt to any changes in the family (27). In the present study, the vast majority of patients with head and neck tumors were young and middle-aged, and patients in this age group are the mainstay of their families; tumor diagnosis and treatment seriously impair their physical function and psychological health, largely affecting the family’s ability to adapt to the disease. Moreover, most of the patients have a disease duration of 1-6 months, which is in the early stage of tumor diagnosis, and it is difficult for the patients to accept the reality, and the psychological pressure is high in this period. Healthcare professionals should encourage patients with head and neck tumors to take the initiative to communicate with family members, express positive or negative emotions within each other, promote the enhancement of family intimacy, make full use of family resources, and improve the level of family adaptability.

Finally, this study uncovered that social support and family resilience acted as chain mediators in the relationship between perceived stress and family adaptability. Social support comes not only from within the family, such as family members and spouses, but also from the external environment, such as friends, neighbors, and social organizations, which provide cancer patients with information about the disease, psychological care, and economic support that are beneficial to alleviate the burden of disease (14, 15, 17). Higher levels of family resilience indicate higher internal and external family strengths and accessible resources, which help cancer patients adapt to crises (18). Furthermore, external family resources can be internalized to improve family adaptability (10, 22). Previous research has also shown that social support is positively correlated with family resilience in oncology patients, and the two promote each other, effectively alleviating the physical, mental, social, and other stresses caused by the disease in individuals and improving their family adaptability (15, 18, 21). It is suggested that medical institutions in a position to do so provide patients with integrated hospital-community-family health-care services, encourage patients to sign up with family doctors to obtain door-to-door services and personalized services throughout the entire process, and set up a health-care consortium to realize the sharing of health-care resources, meet the needs of family members in coping with stress, and achieve the goal of improving the functioning of the family.

## 4 Limitations

This study will certainly have some limitations. Firstly, this was a cross-sectional survey; therefore, the causal link between perceived stress, social support, family resilience, and family adaptability could not be established. To verify the results, more longitudinal investigations are required at a later time. Second, the study was only done at a Chinese institution that specializes in proton heavy ion therapy, with a sample of head and neck cancer patients getting radiotherapy, which may have influenced the results’ universality. Third, this study only looked at cancer patients, not family members, and relying on a single source of data may have influenced the results. As a result, in the future, a binary model should be used to investigate the mechanisms of important variables determining family adaptation. Moreover, this study only looked at how stress perception, social support, and family resilience influence family adaptation. According to the FRRA model, family cohesion, family communication, coping behaviors and positive psychology, and the ability to manage family stress all have an impact on family adaptability, so it is necessary to enrich the research content in the future and thoroughly analyze the influencing factors of family adaptability. Therefore, the promotion of the study’s findings must be done with caution.

## 5 Conclusion

We used a chain-mediated model to verify that not only does perceived stress in head and neck cancer patients receiving radiotherapy directly and negatively predict family adaptability, but also that social support and family resilience play a chain-mediated role in the relationship between perceived stress and family adaptability, suggesting that good social support and higher family resilience can help to reduce patients’ perceived stress of the disease and, in turn, enhance family adaptability. Clinical staff encourage patients to actively communicate with family members, friends, healthcare professionals, and others and provide patients with disease-related knowledge and humanistic care to enrich family resources. At the same time, medical institutions call on social institutions or organizations as much as possible to improve the community service system and the medical insurance system and to give social and economic assistance to the family so as to enhance the family’s adaptability.

## Data Availability

The original contributions presented in the study are included in the article/supplementary material. Further inquiries can be directed to the corresponding author.
